# Protein Inhibitor of Activated STAT (PIAS) Negatively Regulates the JAK/STAT Pathway by Inhibiting STAT Phosphorylation and Translocation

**DOI:** 10.3389/fimmu.2018.02392

**Published:** 2018-10-26

**Authors:** Guo-Juan Niu, Ji-Dong Xu, Wen-Jie Yuan, Jie-Jie Sun, Ming-Chong Yang, Zhong-Hua He, Xiao-Fan Zhao, Jin-Xing Wang

**Affiliations:** ^1^Shandong Provincial Key Laboratory of Animal Cells and Developmental Biology, School of Life Science, Shandong University, Qingdao, China; ^2^State Key Laboratory of Microbial Technology, Shandong University, Qingdao, China

**Keywords:** protein inhibitor of activated STAT, signal transducer and activator of transcription, signaling pathway, antimicrobial peptide, *Marsupenaeus japonicus*

## Abstract

Protein inhibitor of activated STAT (PIAS) proteins are activation-suppressing proteins for signal transducer and activator of transcription (STAT), which involves gene transcriptional regulation. The inhibitory mechanism of PIAS proteins in the Janus kinase (JAK)/STAT signaling pathway has been well studied in mammals and *Drosophila*. However, the roles of PIAS in crustaceans are unclear. In the present study, we identified PIAS in kuruma shrimp *Marsupenaeus japonicus* and found that its relative expression could be induced by *Vibrio anguillarum* stimulation. To explore the function of PIAS in shrimp infected with *V. anguillarum*, we performed an RNA interference assay. After knockdown of *PIAS* expression in shrimp subjected to *V. anguillarum* infection, bacterial clearance was enhanced and the survival rate increased compared with those in the control shrimp (*dsGFP* injection). Simultaneously, the expression levels of antimicrobial peptides (AMPs), including anti-lipopolysaccharide factor (ALF) A1, C1, C2, and CruI-1, increased. Further study revealed that knockdown of *PIAS* also enhanced STAT phosphorylation and translocation. Pulldown assay indicated that PIAS interacts with activated STAT in shrimp. In conclusion, PIAS negatively regulates JAK/STAT signaling by inhibiting the phosphorylation and translocation of STAT through the interaction between PIAS and STAT, which leads to the reduction of AMP expression in shrimp. Our results revealed a new mechanism of PIAS-mediated gene regulation of the STAT signal pathway.

## Introduction

The protein inhibitor of activated STAT (PIAS) family was identified an inhibitor of signal transducer and activator of transcription (STAT) acting on the Janus kinase (JAK)/STAT signaling pathway (1, 2). The PIAS family in mammals has at least five members: PIAS1, PIASxα, PIASxβ, PIAS3, and PIASy. All the PIAS proteins are highly conserved and have common structural features. They have an N-terminal SAF-A/B, Acinus and PIAS (SAP) domain (3), a Pro-Ile-Asn-Ile-Thr (PINIT) motif (4), a RING finger-like Zinc binding domain (RLD) (5), an acidic amino acid domain (AD) (6, 7), and C-terminal Ser/Thr amino acids enriched region (S/T). The SAP domain is involved in sequence- or structure-specific DNA binding (8). The PINIT motif, a highly conserved region of PIAS proteins, is involved in the nuclear retention (4). The RLD is required for the SUMO-E3-ligase activity of PIAS proteins and might be involved in the interaction with other proteins (9). A putative SUMO1-interaction motif (SIM) is present in the AD (10). The C-terminal S/T-rich region is the least conserved and its function remains to be defined.

PIAS has a variety of vital functions, including cell proliferation (11), cell differentiation, cell apoptosis, tumor development, and the immune response (12). The PIAS family can bind to many transcription factors in signaling pathways such as the STAT and nuclear factor kappa B (NF-κB) pathways (13, 14). By interacting with transcription factors or transcriptional coactivators, PIAS could regulate the activity of downstream genes (15).

It has been reported that PIAS1 plays an important role in the negative regulation of the JAK/STAT pathway (16). PIAS proteins inhibit transcription in four ways: closing off the DNA binding activity of transcription factors (2), recruiting cofactor proteins (17), promoting the SUMOylation of transcription factors (18), and sequestering transcription factors in distinct subnuclear structures (19). However, there is little research on the involvement of PIAS in the response to the invasion of pathogenic microorganisms, especially in crustaceans. In the present study, we identified a PIAS cDNA from the kuruma shrimp (*Marsupenaeus japonicus*), and designated its encoded protein as *Mj*PIAS. *MjPIAS* expression was upregulated in shrimp challenged with *Vibio anguillarum*. To analyze the function of *Mj*PIAS in shrimp immunity, RNA interference (RNAi) was performed. After knockdown of *MjPIAS* and *V. anguillarum* infection, the bacterial number in shrimp declined and the shrimp survival rate increased. The possible mechanism of *Mj*PIAS in antibacterial immunity was further studied.

## Materials and methods

### Immune challenge and tissue collection

*M. japonicus* shrimp (weighing 9–11 g/shrimp) were purchased from the seafood market in Jinan, Shandong Province, China. The shrimp were kept at 24°C for 48 h in laboratory tanks at a salinity of 26%0 (w/v) to acclimatize them to the environment. *V. anguillarum* (2 × 10^8^ cells) was injected into the abdomen to infect the shrimp. The same volume of PBS (140 mM NaCl, 2.7 mM KCl, 10 mM Na_2_HPO4, and 1.8 mM KH_2_PO4, pH 7.4) was injected into the control groups. Hemocytes were collected into anticoagulant buffer (450 mM NaCl, 10 mM KCl, 10 mM EDTA, 100 mM HEPES, pH 7.45) after centrifugation at 800 × g for 6 min at 4°C. Other tissues (heart, hepatopancreas, grills, stomach, and intestine) were homogenized separately in TRIpure Reagent (Bioteke, Beijing, China) for RNA extraction or in radio-immunoprecipitation assay (RIPA) buffer (50 mM Tris-HCl, 150 mM NaCl, 0.1% SDS, 0.5% Nonidet P-40, 1 mM EDTA, 0.5 mM PMSF, pH 7.5) for protein extraction. The supernatant was obtained by centrifugation at 12,000 × g for 10 min at 4°C.

### RNA extraction and cDNA reverse transcription

Total RNA was first extracted from the six tissues (five organs plus the hemocytes) using the Trizol reagent and then reverse transcribed to cDNA according to the SMART cDNA reverse transcription kit (M-MLV version; Takara, Dalian, China) using the primers Smart F and oligo anchorR (Table 1).

**Table 1:** Sequences of the primers used in this study.

**Primer**	**Sequence (5^′^–3^′^)**
Smart F	TACGGCTGCGAGAAGACGACAGAAGGG
Oligo anchorR	GACCACGCGTATCGATGTCGACT16(A/C/G)
*Mj*PIAS-RT-F	ATCGCCACCACTTCCTTG
*Mj*PIAS-RT-R	CCTCTGATTTCCGCTTCTC
*Mj*PIAS-RLD-EXF	TACTCAGGATCCCTGACCTCAGATGAC
*Mj*PIAS-RLD-EXR	TACTCACTCGAGGAAGTATCCATCAAT
Actin-RT-F	AGTAGCCGCCCTGGTTGTAGAC
Actin-RT-R	TTCTCCATGTCGTCCCAGT
*Mj*PIAS-Ri-F	GCGTAATACGACTCACTATAGGAAGTGATCTGCTTAGACA
*Mj*PIAS-Ri-R	GCGTAATACGACTCACTATAGGCAAATGCTGCAACCATCC
GFP-RNAi-F	TAATACGACTCACTATAGGGGGGTGGTCCCAATTCTCGTGGAAC
GFP-RNAi-R	TAATACGACTCACTATAGGGCTTGTACAGCTCGTCCATGC
ALF-A1-RT-F	CTGGTCGGTTTCCTGGTGGC
ALF-A1-RT-R	CCAACCTGGGCACCACATACTG
ALF-C1-RT-F	CGCTTCAAGGGTCGGATGTG
ALF-C1-RT-R	CGAGCCTCTTCCTCCGTGATG
ALF-C2-RF-F	TCCTGGTGGTGGCAGTGGCT
ALF-C2-RF-R	TGCGGGTCTCGGCTTCTCCT
CruI-1-RT-F	TGCTCAGAACTCCCTCCACC
CruI-1-RT-R	TTGAATCAGCCCATCGTCG

### cDNA cloning of *MjPIAS* and phylogenetic analysis

The sequence of *MjPIAS* was obtained by transcriptomic sequencing of hemocytes, and confirmed by replication with reverse transcription PCR (RT-PCR) using specific primers (Table 1). The EXPASY translation tool was used to analyze the deduced amino acid sequence (http://web.expasy.org/translate/). The domain architecture was predicted using SMART (http://smart.embl.de/). The sequences of PIAS from other species were collected from NCBI GenBank (http://www.ncbi.nlm.nih.gov/genbank/). A phylogenetic tree was constructed using MEGA version 5.0.

### Tissue distribution and expression profile

The tissue distribution of *MjPIAS* was determined using semi-quantitative RT-PCR with primers *Mj*PIAS-RT-F and *Mj*PIAS-RT-R (Table 1). The actin mRNA was amplified using primers Actin-RT-F and Actin-RT-R as an internal control. The RT-PCR procedure was performed as follows: 94°C for 3 min; 26 cycles of 94°C for 20 s, 54°C for 20 s, 72°C for 20 s; and a final step of 72°C for 10 min. The expression profile was further assessed using quantitative real-time PCR (qPCR) as follows: 94°C for 10 min, 40 cycles of 94°C for 15 s and 60°C for 1 min; followed by reading at 78°C for 2 s. Each experiment was repeated three times and results were calculated using the 2^−ΔΔ*Ct*^ method. An unpaired *t-*test was used for statistical analysis and a significant difference was accepted at *p* < 0.05.

### RNA interference and bacterial clearance assay

Double-stranded RNA (*dsMjPIAS*) was produced to knockdown the expression of *MjPIAS*. The *PIAS* fragment was amplified using primers *Mj*PIAS-Ri-F and *Mj*PIAS-Ri-R, and then used to produce the dsRNA via T7 RNA polymerase (Fermentas, Burlington, Canada). The green fluorescent protein (GFP) was used as a control to synthesis GFP dsRNA. DsRNA (30 μg) was injected into shrimp and an additional 30 μg dsRNA dose was injected 24 h later. Interference effects were detected at 48 h using qRT-PCR following *V. anguillarum* challenge (2 × 10^8^ cells). Bacterial clearance assays were performed 3 h after *V. anguillarum* injection. The shrimp cell-free hemolymph was collected and gradient diluted to 200-fold. The diluted hemolymph was smeared onto 2216E-agar culture medium and the number of bacterial colonies was counted on the second day.

### Survival rate

To investigate the effect of PIAS *in vivo*, a survival rate assay was performed after RNAi of the *PIAS* gene following *V. anguillarum* challenge (30 μl, 2 × 10^8^ cells) at 48 h post dsRNA injection; the *dsGFP*-injection group served as the control. The number of dead shrimp was counted every half day, and the cumulative survival rates were calculated using GraphPad Prism 5.0. The experiment was repeated three times.

### Immunocytochemical analysis to detect the STAT subcellular location in hemocytes

To detect whether PIAS inhibits STAT transcriptional activity in hemocytes, we performed immunocytochemistry. *V. anguillarum* (2 × 10^8^ cells) was injected into shrimp after knockdown of *MjPIAS* for 48 h. Hemocytes were collected 3 h post infection and spread on a glass slide. Immunocytochemistry was performed following a previously described method (20) with an anti-STAT antibody (prepared in our laboratory) and an anti-p-STAT antibody (Abcam, San Francisco, USA) (21). All glass slides were observed under a fluorescence microscope (Olympus BX51, Tokyo, Japan).

### Western blotting

The hemolymph was extracted into anticoagulant buffer and centrifuged at 800 × g for 6 min at 4°C for hemocyte collection, which were resuspended in RIPA buffer. Each sample was separated by 12.5% SDS-PAGE and transferred onto a nitrocellulose membrane. After blocking with 3% non-fat milk in Tris-buffered saline (TBS) (150 mM NaCl, 3 mM EDTA, 50 mM Tris-HCl, pH 8.0) for 1 h, the membrane was incubated with anti-*Mj*STAT (1/200 diluted in 3% blocking milk solution) overnight at 4°C. The membrane was then washed three times with TBST buffer (150 mM NaCl, 3 mM EDTA, 0.1% Tween-20, 50 mM Tris-HCl, pH 8.0) and incubated with horseradish peroxidase (HRP)-conjugated goat anti-rabbit antibody (1/10,000 diluted in 3% blocking milk solution) (ZSGB Bio, Beijing, China) for 3 h at room temperature. After washing with TBST three times and with TBS three times, the immunoreactive proteins on the membrane were visualized using reaction media (1 ml 4-chloro-1-naphthol, 6 μl H_2_O_2_, 9 ml TBS) in the dark.

### Protein recombinant expression and purification

*Mj*PIAS-RLD fragments were amplified using specific primers *Mj*PIAS-RLD-EXF/R. Then the fragments and pGEX4T-1 plasmid were digested with the same restriction enzymes (*Bam*HI and *Xho*I). Afterwards, the fragments were subcloned into pGEX4T-1 plasmid. The recombinant plasmids were transformed into *Escherichia coli* Rosseta (DE3) cells for expression with 0.5 mM isopropyl-β-D-thiogalactopyranoside (IPTG). Soluble *Mj*PIAS-RLD proteins were purified using GST-Bind resin (GenScript, Nanjing, China) affinity chromatography.

The His-tagged *Mj*STAT plasmid (pET-32a/*Mj*STAT) in *E. coli* constructed in our laboratory before was used for *Mj*STAT expression. *Mj*STAT expressed as inclusion bodies. Buffer A (50 mM Tris-HCl, 5 mM EDTA, pH 8.0) and Buffer B (50 mM Tris-HCl, 5 mM EDTA, 2 M urea, pH 8.0) reagents were used for washing the inclusion bodies. Then the inclusion bodies were dissolved in denaturing solution (0.1 M Tris-HCl, 10 mM DL-Dithiothreitol, 8 M urea) and refolded in TBS buffer for purification and pull-down experiment.

### Pull-down assay

GST-tagged *Mj*PIAS-RLD (200 μg) was incubated with His-tagged *Mj*STAT(with a 1:1 ratio) at 4°C overnight. Then the GST∂Bind resin (50 μl) was added and incubated for another 45 min. After washing with PBS five times, elution buffer (10 mM reduced glutathione, 50 mM Tris-HCl, pH 8.0) was added for elute out the bound proteins. The mixture was analyzed by SDS-PAGE.

We further use the recombinant *Mj*PIAS to pull down native STAT in shrimp. Shrimp was challenged by *V. anguillarum* for 3 h and hemocytes were extracted for protein extraction. GST-tagged *Mj*PIAS-RLD proteins (200 μg) were incubated with natural protein (homogenate of hemocytes, 1 mg) at 4°C overnight. The following steps are the same as above. The resulting mixture was separated by SDS-PAGE, and then transferred onto a nitrocellulose membrane for Western blotting with anti-STAT. GST proteins served as control.

### Statistical methods

The data are presented as the mean ± SD derived from at least three independent tests. The difference was determined by one-way ANOVA, followed student *t*-test. A significant difference was accepted at *p* < 0.05.

### Ethics statement

All animal-involving experiments of this study were approved by the Ethics Committee of School of Life Sciences, Shandong University, and all efforts were made to minimize suffering.

## Results

### Bioinformatic analysis of *Mj*PIAS

**Figure 1:**
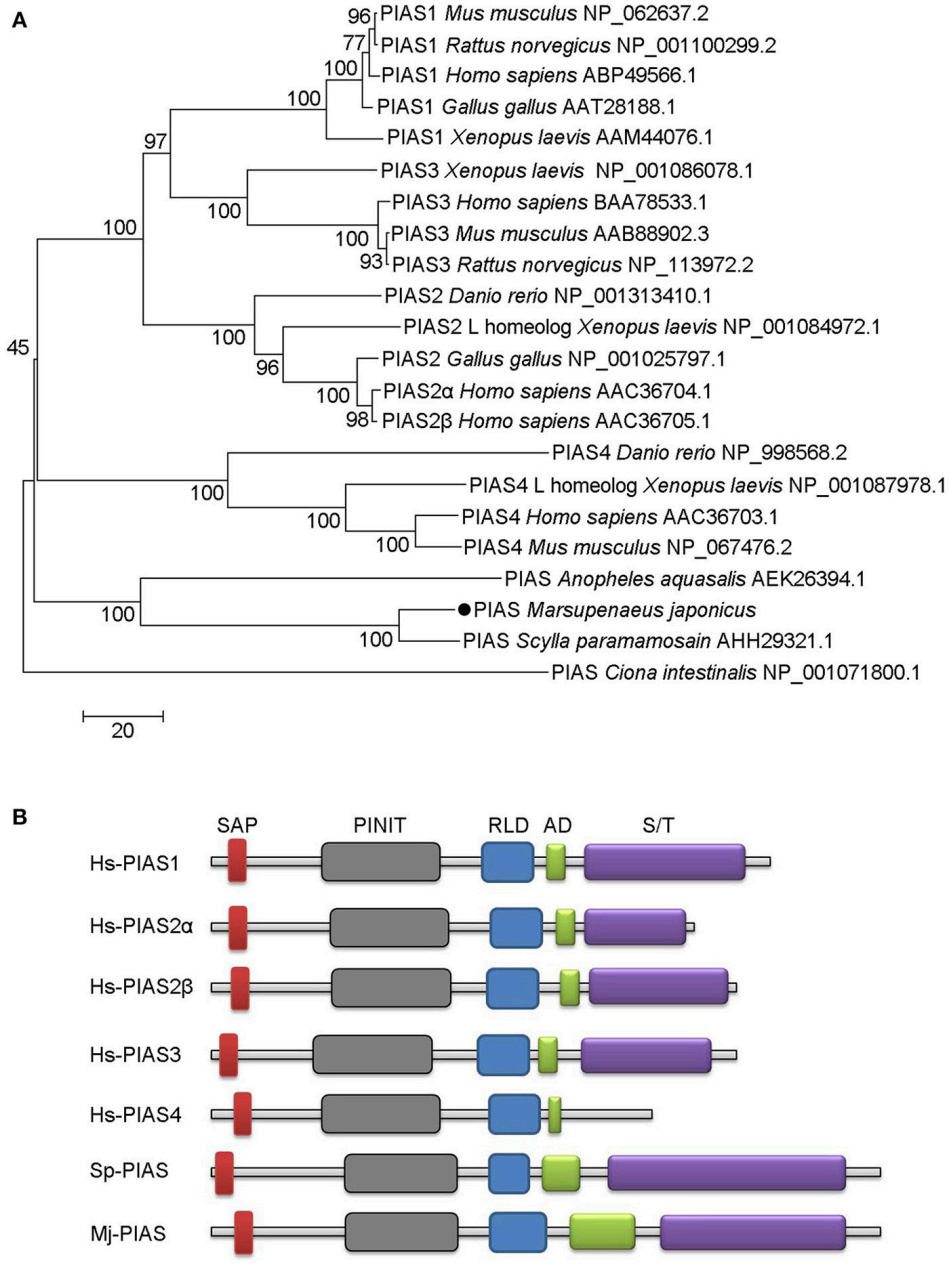
Phylogenetic tree and domain architectures of PIAS from shrimp and other species. **(A)** Phylogenetic tree of PIAS proteins. The full-length amino acid sequences of PIAS proteins from different species were collected from GenBank and the neighbor-joining phylogenetic tree was constructed using MEGA version 5.0 software. The shrimp PIAS is marked with a dot. The scale bar is shown to indicate the branch length. **(B)** Domain architectures of PIAS proteins. Hs, *Homo sapiens*; Sp, *Scylla paramamosain*; Mj, *Marsupenaeus japonicus*. Hs-PIAS1, ABP49566.1; Hs-PIAS2α, AAC36704.1; Hs-PIAS2β, AAC36705.1; Hs-PIAS3, BAA78533.1; Hs-PIAS4, AAC36703.1; Sp-PIAS, AHH29321.1.

We obtained the full-length cDNA sequence of *Mj*PIAS from *M. japonicas* (GenBank accession no. MH238442). It contains a SAP, PINIT, RLD, highly acidic region domain (AD), and S/T domains. Phylogenetic analysis revealed that PIAS from *M. japonicas* is closely related to that from *Scylla paramamosain* (Figure 1A). We also analyzed the domain architecture of PIAS proteins from different species and found that they have the similar domains (Figure 1B), including the SAP domain, which is located at the N-terminus of PIAS proteins, the PINIT domain, an RLD, an AD, and an S/T domain in the C-terminal region of the PIAS proteins.

### *Mj*PIAS was upregulated in shrimp when challenged by bacteria

**Figure 2:**
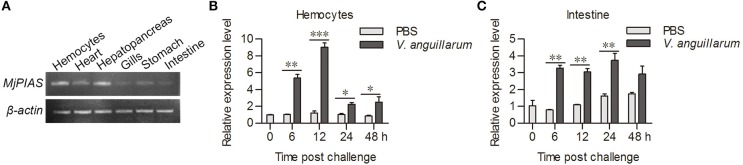
*MjPIAS* was upregulated in shrimp after *V. anguillarum* challenge. **(A)** Semi-quantitative RT-PCR analysis of PIAS transcription in different tissues. beta-Actin was used as the control. **(B,C)** Temporal expression patterns of *MjPIAS* in hemocytes **(B)** and intestines **(C)** of shrimp after injection of *V. anguillarum*. PBS was used as the control. Three repeats were performed and the data were analyzed using an unpaired *t-*test. The asterisks indicate significant differences *vs*. the control (*p < 0.05, **p < 0.01, ***p < 0.001).

We used RT-PCR to analyze the distribution of *MjPIAS* in different tissues at RNA level. *MjPIAS* mRNA was expressed in hemocytes and in all five tested organs (heart, hepatopancreas, gills, stomach, and intestine), with high expression in the hemocytes and hepatopancreas (Figure 2A). We infected shrimp with *V. anguillarum* and extracted the RNA of hemocytes and intestines at different time points. The expression profiles were detected using qRT-PCR. The result showed that *MjPIAS* expression was significantly upregulated from 6 to 48 h post bacterial injection, and reached its highest level at 12 h in the hemocytes (Figure 2B). The high expression of *MjPIAS* in the intestines continued from 6 to 24 h (Figure 2C). These results suggested that *Mj*PIAS might be associated with antibacterial immunity in shrimp.

### PIAS is involved in antibacterial immunity

**Figure 3:**
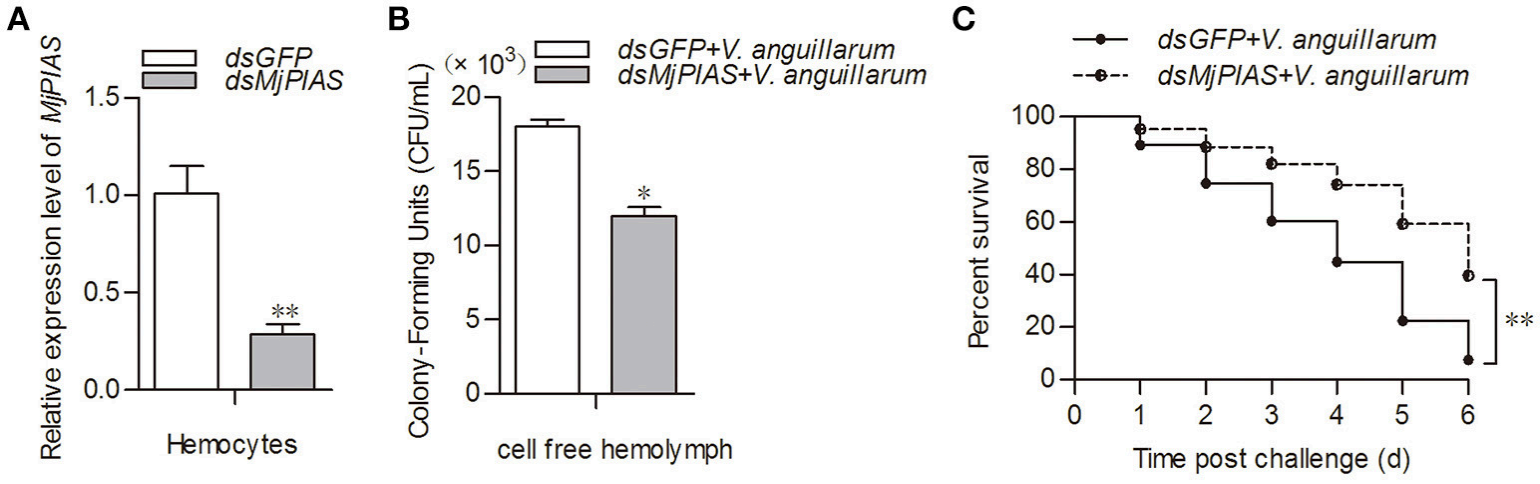
Knockdown of *MjPIAS* in shrimp facilitated antibacterial immunity in shrimp. **(A)** Efficiency of *MjPIAS* RNA interference was analyzed using qRT-PCR. **(B)** Bacterial clearance was detected after *V. anguillarum* challenge in the *dsGFP* and *dsMjPIAS* injection groups. **(C)** Survival rates of *MjPIAS*-knockdown shrimp and the *dsGFP* injection group. The dsRNA was injected into the different groups, followed by injection of *V. anguillarum* at 48 h post *dsRNA* injection. The data were analyzed using the unpaired *t-*test. The asterisks indicate significant differences between the *dsGFP* and *dsMjPIAS* injection groups (*p < 0.05, **p < 0.01).

To study the function of *Mj*PIAS, we performed an RNA interference assay to knockdown *MjPIAS* expression in shrimp. Following *V. anguillarum* infection, we detected the bacterial number *in vivo* and the shrimp survival rate. *MjPIAS* expression was successfully knocked down in hemocytes at 48 h after the injection of *dsMjPIAS* (Figure 3A). The number of colony-forming units (CFUs) was significantly reduced in the *dsPIAS* group compared with that in the *dsGFP* injection group (Figure 3B). Meanwhile, the survival rate of *MjPIAS*-knockdown-shrimp was significantly higher than that of the *dsGFP* injection group (Figure 3C). These results suggested that knockdown of *MjPIAS* enhanced the shrimp's antibacterial ability. In other words, *Mj*PIAS inhibits the antibacterial ability of shrimp.

### *Mj*PIAS inhibits the expression of *AMP* genes

**Figure 4:**
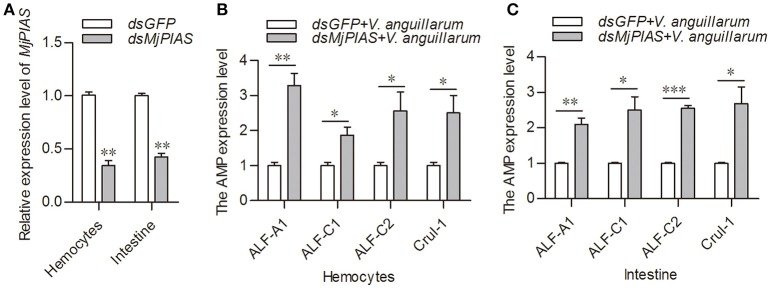
Expression of antimicrobial peptides was increased in the *MjPIAS*-knockdown shrimp. **(A)** Efficiency of *MjPIAS* RNAi. **(B,C)** After the injection of dsRNA for 48 h, bacterial inoculation was performed and qRT-PCR was used to detect expression of four AMPs (*ALF-A1, ALF-C1, ALF-C2*, and *CruI-1*). Three repeats were performed and the data were analyzed using the unpaired *t-*test. The asterisks indicate significant differences between the RNAi and control groups (*p < 0.05, **p < 0.01, ***p < 0.001).

The initial description of the function for PIAS proteins was as negative regulators of STATs (22). In our previous study, we showed that activation of the JAK/STAT pathway resulted in AMPs expression (21). To explore whether the antibacterial inhibition ability of *Mj*PIAS is caused by the negative regulation of STAT in shrimp, we detect the expression levels of four AMPs (ALF-A1, ALF-C1, ALF-C2, and CruI-1) that are regulated by the JAK/STAT pathway (21) in *MjPIAS*-knockdown shrimp followed by *V. anguillarum* injection. *MjPIAS* expression was knocked down in hemocytes at 48 h (Figure 4A). After the injection of *V. anguillarum*, the expression levels of AMPs were detected, and the results showed their expression levels were markedly increased compared with those in the control (*dsGFP* injection) in the hemocytes (Figures 4B,C). These results suggested that *Mj*PIAS inhibits shrimp's antibacterial ability by negatively regulating the expression of AMPs controlled by the JAK/STAT pathway.

### PIAS inhibits STAT phosphorylation and translocation

**Figure 5:**
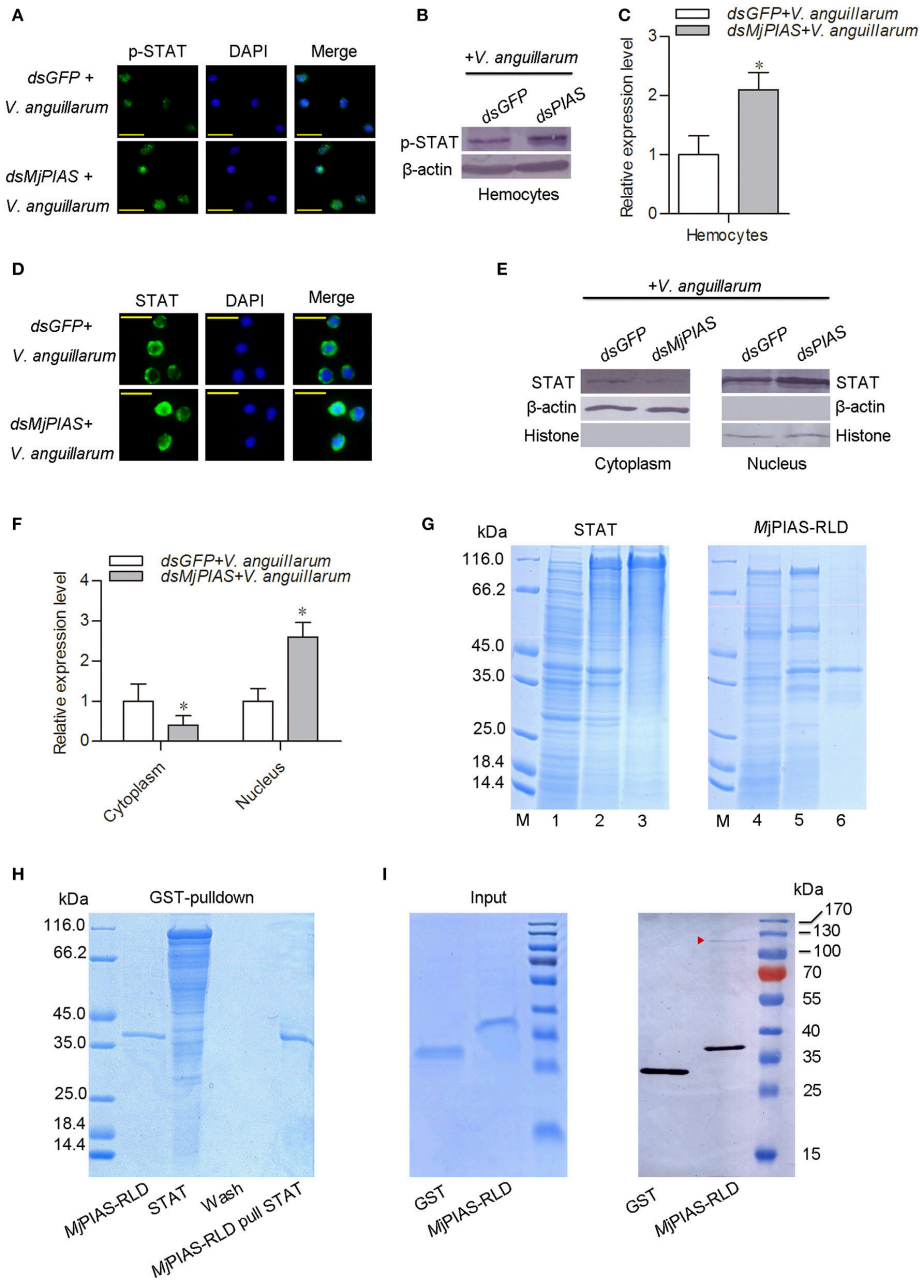
*PIAS* knockdown promotes STAT translocation and phosphorylation in hemocytes. **(A)** An immunocytochemical assay was performed to detect the phosphorylated (p)-STAT in the hemocytes of *MjPIAS*-RNAi shrimp. **(B)** Proteins were extracted from hemocytes to detect the STAT subcellular location in *MjPIAS*-knockdown and control shrimp using western blotting. **(C)** Statistical analysis of the western blotting results shown in **(B)**. **(D)** Immunocytochemical assay to detect STAT translocation in dsRNA-injection shrimp followed *V. anguillarum* injection. **(E)** Total cytoplasmic and nuclear proteins were extracted from hemocytes of *MjPIAS-RNAi* shrimp and the control group, respectively. STAT was detected by western blotting using anti-STAT antibodies. **(F)** Statistical analysis of the western blotting results shown in **(E)** (*p < 0.05). **(G)** Recombinant expression and purification of STAT and *Mj*PIAS-RLD. Lane M. Protein standards; Lane 1, total proteins from *E. coli* with pET-32a/STAT without IPTG induction; lane 2, total proteins from the *E. coli* with IPTG induction; lane 3, purified recombinant STAT; Lane 4, total proteins from *E. coli* with pGEX4T1-*Mj*PIAS-RLD without IPTG induction; lane 5, total proteins from the *E. coli* with IPTG induction; lane 6, purified recombinant *Mj*PIAS-RLD. **(H)** The interaction of r*Mj*PIAS-RLD with r*Mj*STAT analyzed using GST-Pulldown assay. **(I)** Interaction between *Mj*PIAS-RLD with activated STAT from hemocytes of shrimp challenged by *V. anguillarum*. Same amount of proteins of GST and GST-tagged *Mj*PIAS-RLD were shown at the input picture (left). The recombinant proteins could interact with activated STAT was analyzed using western blotting. The red triangle shows the STAT.

To analyze the molecular mechanism whereby *Mj*PIAS negatively regulates transcription of AMPs, the translocation and phosphorylation of STAT were detected by immunocytochemistry (Figure 5A) and western blotting (Figures 5B,C) using p-STAT antibody, and results showed that the phosphorylation of STAT was enhanced in hemocytes after *V. anguillarum* injection in the *MjPIAS*-silenced shrimp. The immunocytochemical assay using STAT antibody was also performed and result showed that STAT translocation into the nucleus increased in the *MjPIAS*-knockdown shrimp (Figure 5D). Meanwhile, we extracted the cytoplasmic and nuclear proteins from the hemocytes of *PIAS-RNAi* and control shrimp. The level of STAT in the cytoplasm and nucleus was detected using anti-STAT antibodies (Figures 5E,F). The results showed that much more STAT was detected in nucleus in the *MjPIAS*-RNAi shrimp comparing with control. These results suggested that *MjPIAS* negatively regulates STAT by inhibiting its translocation and phosphorylation.

### *Mj*PIAS interacts with the native STAT of shrimp

In order to detect the interaction between *Mj*PIAS and STAT, recombinant *Mj*PIAS-RLD and STAT were purified from *E. coli* (Figure 5G). GST-pulldown was performed and there was no interaction was identified between the two recombinant proteins (Figure 5H).The recombinant *Mj*PIAS-RLD was further used to pull down native STAT and result showed that *Mj*PIAS-RLD could interact with native STAT from hemocytes of shrimp challenged by *V. anguillarum* (Figure 5I). These results suggested that *Mj*PIAS could interact with activated STAT.

## Discussion

In this study, we identified *Mj*PIAS, which was determined to negatively regulate STAT by analyzing the AMP transcription regulated by the JAK/STAT pathway in kuruma shrimp. Further study found that the molecular mechanism of the *Mj*PIAS function comprised inhibiting STAT translocation and phosphorylation, and further inhibiting the expression of AMPs.

PIAS proteins play important regulatory role in cytokine signaling and immune system (12, 23). PIAS proteins act as transcriptional co-regulators of at least 60 different proteins, and either repress or activate their transcription. PIAS proteins interact with several transcription factors, such as STAT, NF-κB, Jun, and p53, many of which are key transcription factors involved in the immune system. STAT is an important transcription factor in the JAK/STAT signaling pathway (24–26). STAT is activated in the cytoplasm and forms dimers, resulting in its translocation to the cell nucleus where it combines with the corresponding cis-elements (27), activating downstream gene transcription. In shrimp, the JAK/STAT pathway can be directly activated by bacterial infection, which regulates the expression of AMPs, including ALF-A1, ALF-C1, ALF-C2, CruI-1, and CruI-5 (21). Previous studies showed that PIAS could regulate the activity of the downstream genes by interaction with transcription factors or other transcriptional coactivators (25, 28, 29). Based on its similar structural characteristics to PIAS proteins from other species, we explored whether *Mj*PIAS could negatively regulate the shrimp JAK/STAT signaling pathway. Shrimp were challenged with *V. anguillarum* after the knockdown of *MjPIAS*, which resulted in increased expression of AMPs regulated by the JAK/STAT signaling pathway. In the bacterial clearance assay, the *dsPIAS* group showed stronger antibacterial ability. In addition, the survival experiment showed that the *dsPIAS* group had a higher survival rate compared with that of the *dsGFP* group. Taken together, the results indicated that PIAS is a negative regulator of the JAK/STAT pathway.

**Figure 6:**
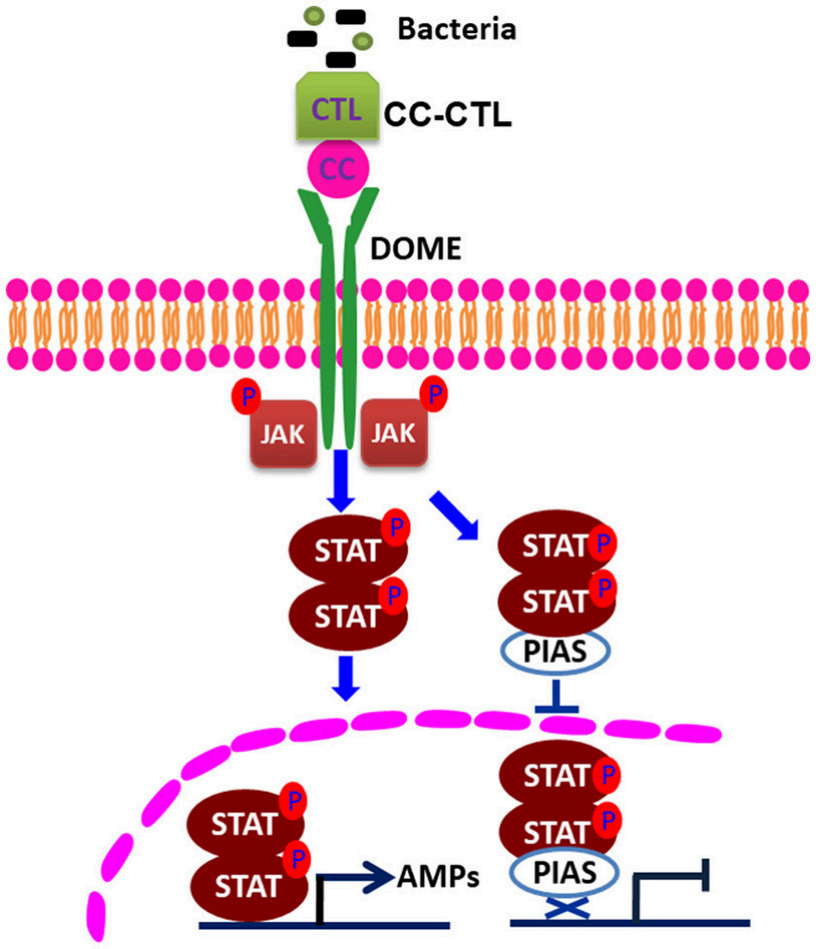
Schematic representation of the mechanism of action of PIAS in the regulation of the STAT signaling pathway. C-type lectin, with CTL and CC domains, recognizes bacteria and interacts with Domelee, and the JAK/STAT signaling pathway is activated. JAK phosphorylates STAT. The phosphorylated STAT enters the nucleus and binds to the promoters of AMP genes to regulate their transcriptions (21). PIAS negatively regulates STAT by inhibiting STAT phosphorylation and nuclear translocation in the JAK/STAT signaling pathway.

Several studies have shown that PIAS proteins play important roles in the regulation of the STAT signaling pathway. PIAS family members interact with various STATs in mammals, and the interaction requires cytokine stimulation (23). PIAS interacts with the dimeric form, but not the monomeric form, of STAT (30). In our pulldown study with *Mj*PIAS with STAT, we found that recombinant *Mj*PIAS could not interact with recombinant STAT. However, recombinant *Mj*PIAS could interact with native STAT from bacterial challenged shrimp (Figures 5H,I). These results suggest that *Mj*PIAS interacts with dimeric form of STAT (activated STAT) in shrimp. The binding of PIAS to STAT results in inhibition of STAT-mediated gene activation by blocking the DNA binding activity of STAT (22), by recruiting other co-repressor molecules, such as HDACs, to inhibit transcription (31), and by promoting the sumoylation of a transcription factor (5), and by sequestering transcription factors in certain subnuclear structures that are enriched for corepressor complexes (32). Although the majority of reported interactions of PIAS proteins occurred with transcription factors or other proteins linked to nuclear regulation (33), there are examples of cytoplasmic interactions in which PIAS proteins are involved, such as interaction of septins with PIAS ortholog Siz1 in yeast bud neck (34), and functional interaction of PIAS3 with kainate receptor subunit, glutamate receptor-8 (35). In the present study, we found that the phosphorylation of STAT was enhanced in hemocytes after *V. anguillarum* injection in the *MjPIAS*-silenced shrimp by immunocytochemistry and western blotting using p-STAT antibody (Figures 5A–C). We also detected the level of STAT in the cytoplasm and nucleus of the hemocytes from *PIAS-RNAi* and control shrimp using anti-STAT antibodies, and found that much more STAT was detected in nucleus in the *MjPIAS*-RNAi shrimp comparing with control (Figures 5E,F). All the results revealed that *Mj*PIAS might interact with STAT at cytoplasm and inhibit its translocation into the nucleus in addition to interaction with STAT in nucleus. These data suggested that *Mj*PIAS negatively regulates STAT by inhibiting its translocation and phosphorylation. This is a new molecular mechanism for the negative regulation of transcription by PIAS, comprising inhibiting STAT phosphorylation and translocation into nucleus. Taken together with the results of our former study concerning the function of the JAK/STAT signal pathway (21), we hypothesized the possible mechanism of action of *Mj*PIAS (Figure 6). The C-type lectin, *Mj*CC-CL recognizes infected bacteria, and interacts with the receptor Dome, and activates JAK/STAT signal pathway. The transcription factor STAT is phosphorylated by JAK, and translocates into the nucleus, inducing the AMP expression. The pathway is subtly regulated by *Mj*PIAS in cytoplasm and nucleus by interaction with STAT, and inhibiting STAT phosphorylation and translocation. To the best of our knowledge, this is the first report of the mechanism of PIAS-mediated gene regulation in the STAT signaling pathway in animals.

## Author contributions

J-XW and G-JN designed the experiments and wrote the manuscript. G-JN performed the majority of the experiments and analyzed data. J-DX, W-JY, J-JS, M-CY, and Z-HH contributed experimental suggestions. X-FZ helped to design the experiments. All authors revised the manuscript.

### Conflict of interest statement

The authors declare that the research was conducted in the absence of any commercial or financial relationships that could be construed as a potential conflict of interest.
